# Outcome of COVID19 in Patients With Osteogenesis Imperfecta: A Retrospective Multicenter Study in Saudi Arabia

**DOI:** 10.3389/fendo.2021.800376

**Published:** 2022-01-13

**Authors:** Abeer N. Alshukairi, Hazem Doar, Afaf Al-Sagheir, Mona A. Bahasan, Anas A. Sultan, Mohammad K. Al Hroub, Dina Itani, Imran Khalid, Mohammed F. Saeedi, Sarah Bakhamis, Laila Layqah, Afnan A. Almutairi, Mona Saifullah, Lama Hefni, Awad Al-Omari, Basem M. Alraddadi, Salim A. Baharoon

**Affiliations:** ^1^ Department of Medicine, King Faisal Specialist Hospital and Research Centre, Jeddah, Saudi Arabia; ^2^ College of Medicine, AlFaisal University, Riyadh, Saudi Arabia; ^3^ Department of Surgery, King Faisal Specialist Hospital and Research Centre, Jeddah, Saudi Arabia; ^4^ Department of Pediatrics, King Faisal Specialist Hospital and Research Centre, Riyadh, Saudi Arabia; ^5^ Department of Pediatrics, King Faisal Specialist Hospital and Research Centre, Jeddah, Saudi Arabia; ^6^ Department of Infection Control and Hospital Epidemiology, King Faisal Specialist Hospital and Research Centre, Jeddah, Saudi Arabia; ^7^ Department of Family Medicine, King Faisal Specialist Hospital and Research Centre, Jeddah, Saudi Arabia; ^8^ Research Office, King Abdullah International Medical Research Centre, Riyadh, Saudi Arabia; ^9^ Department of Critical Care, Dr Sulaiman Al Habib Medical Group, Riyadh, Saudi Arabia; ^10^ Department of Critical Care, King Abdulaziz Medical City, Riyadh, Saudi Arabia

**Keywords:** outcome, bisphosphonate, COVID - 19, fracture, osteogenesis imperfecta

## Abstract

**Background:**

Although genetic diseases are rare, children with such conditions who get infected with COVID-19 tend to have a severe illness requiring hospitalization. Osteogenesis imperfecta (OI) is a rare genetic disorder of collagen resulting in fractures and skeletal deformities. Kyphoscoliosis, restrictive lung disease, and pneumonia worsen the prognosis of patients with OI. The use of bisphosphonate improves bone mineral density (BMD) and reduces fractures in OI. There is no literature describing the impact of COVID-19 in patients with OI.

**Methodology:**

A retrospective multi-center study was performed in three hospitals in Jeddah and Riyadh, Saudi Arabia, from March 1st, 2020, until August 31st, 2021, aiming to evaluate the outcome of COVID-19 in patients with OI. Demographics, vaccination status, underlying kyphoscoliosis, functional status, use of bisphosphonate, BMD, and COVID-19 severity, and course were recorded for all patients.

**Results:**

Twelve cases of confirmed COVID-19 were identified among 146 patients with OI. 9 (75%) of patients were less than 18 years, 6 (50%) were male, 5 (41%) had kyphoscoliosis, and 5 (41%) were wheelchair-bound. 6 (50%) received bisphosphonate, and 7(58%) had normal BMD. All patients had mild disease and did not require hospitalization. None of OI the patients with COVID-19 were fully vaccinated before the infection, and some were ineligible for vaccination.

**Conclusion:**

Patients with OI and COVID-19 in our study recovered without complications, unlike patients with other genetic diseases. Young age and mild illness contributed to the favorable outcome. Half of the patients received bisphosphonate and had normal BMD.

## Introduction

As the COVID-19 pandemic continues to cause new cases worldwide, the epidemiology of SARS-CoV-2 infection is evolving with the emergence of variant strains of concern, in addition to the reporting of an increasing number of cases among children (1, 2). Both adults and children with comorbidities infected with COVID-19 are at risk to develop severe pneumonia requiring hospitalization (3, 4). Although they are rare, children with genetic diseases infected with SAS-CoV-2 infection were more likely to be hospitalized and develop severe illness and intensive care unit admission (5). Studies evaluating the outcome of COVID-19 in adults in Saudi Arabia showed that patients with comorbidities, including diabetes mellitus and cardiovascular diseases, had a severe illness and high mortality (6, 7). The mortality rate among diabetic COVID-19 patients was 20.3% compared to 12.3% in non-diabetic COVID-19 patients (6). On the other hand, studies describing the outcome of COVID-19 in children in Saudi Arabia were variable depending on the study design, disease severity, and inclusion of patients with comorbidities. While studies that included hospitalized COVID-19 children reported 4% mortality among patients requiring intensive care admission (8, 9), studies that included non-hospitalized COVID-19 children described favorable outcomes (10, 11). In the two most extensive studies of COVID-19 in children in Saudi Arabia, only 15% of children had comorbidities (10, 11).

Osteogenesis Imperfecta (OI) is a rare genetic disease that affects type I collagen with variable severity resulting in skeletal abnormalities and variable predisposition to fractures. Based on the genetic classification of OI, mild type I disease was caused by a deficiency of normal collagen. In contrast, lethal type II, severe type III, and moderate type IV diseases had abnormal collagen structure (12). Scoliosis, restrictive lung disease and respiratory tract infections significantly impact the quality of life of patients with OI and are considered important causes of death in a severe form of OI (13). Data on outcomes of OI patients infected with SARS-CoV-2 is limited. Among a cohort of 146 OI patients, we described the favorable outcome of 12 COVID-19 patients and evaluated various factors contributing to their excellent prognosis.

## Materials and Methods

We performed a retrospective study to identify all cases of OI diagnosed and followed in three tertiary care centers in Jeddah and Riyadh in Saudi Arabia. We reviewed their medical records for demographics, functional status, kyphoscoliosis, results of pulmonary function tests, use of calcium, vitamin D, and bisphosphonate, and effects of bone mineral density. We also checked their vaccination status, the type of vaccine, the time of vaccination, and COVID 19 diagnosis within the last 18 months from the Ministry of Health database. Patients with OI confirmed the home isolation clinic nurses called COVID-19 to evaluate COVID-19 disease severity, hospitalization, and outcome. Patients with OI and infected with COVID-19 were included from March 1st, 2020, until August 31st, 2021. The institutional research board approved our study of King Faisal Specialist Hospital and Research Center in Jeddah, Saudi Arabia (IRB-2021-CR-17).

## Results

### Description of the OI Cohort Study

146 patients with OI were included in our study. 119 (82%) were less than 18 years old, with a mean age of 12.9. 97 (66%) were male. 35 (24%) had kyphoscoliosis. 92 (63%) of patients were receiving bisphosphonate. 83 (57%) were vaccine eligible. 55/83 (66%) of vaccine-eligible patients were fully vaccinated. 58/67 (87%) of patients received Pfizer-BioNTech vaccination. 12/146 (8%) of patients with OI had confirmed COVID-19 based on positive SARS-CoV-2 nasopharyngeal PCR (**Table 1**).

**Table 1:** Characteristics of patients with Osteogenesis Imperfecta (n = 146).

Parameter	Value, n (%)
Age (mean ± SD)	(12.9 ± 6.6)
Adult >18 years old	27 (18)
Child < 18 years old	119 (82)
Gender	
Male	97 (66)
Female	49 (34)
Patients infected with COVID 19 within the last 18 months	12 (8)
Patients with underlying Kyphoscoliosis	35 (24)
Patients eligible for vaccination	83 (57)
Vaccinated amongst eligible patients	
Full	55 (66)
Partial	12 (15)
None	16 (19)
Type of vaccine amongst vaccinated patients	
Pfizer-BioNtech	58 (87)
Oxford-AstraZeneca	8 (12)
Moderna	1 (1)
Bisphosphonate usage	
Pamidronate	16 (11)
Zoledronate	76 (52)
None	54 (37)

### Description of the COVID-19 Case Series Among Patients With OI

**Figure 1:**
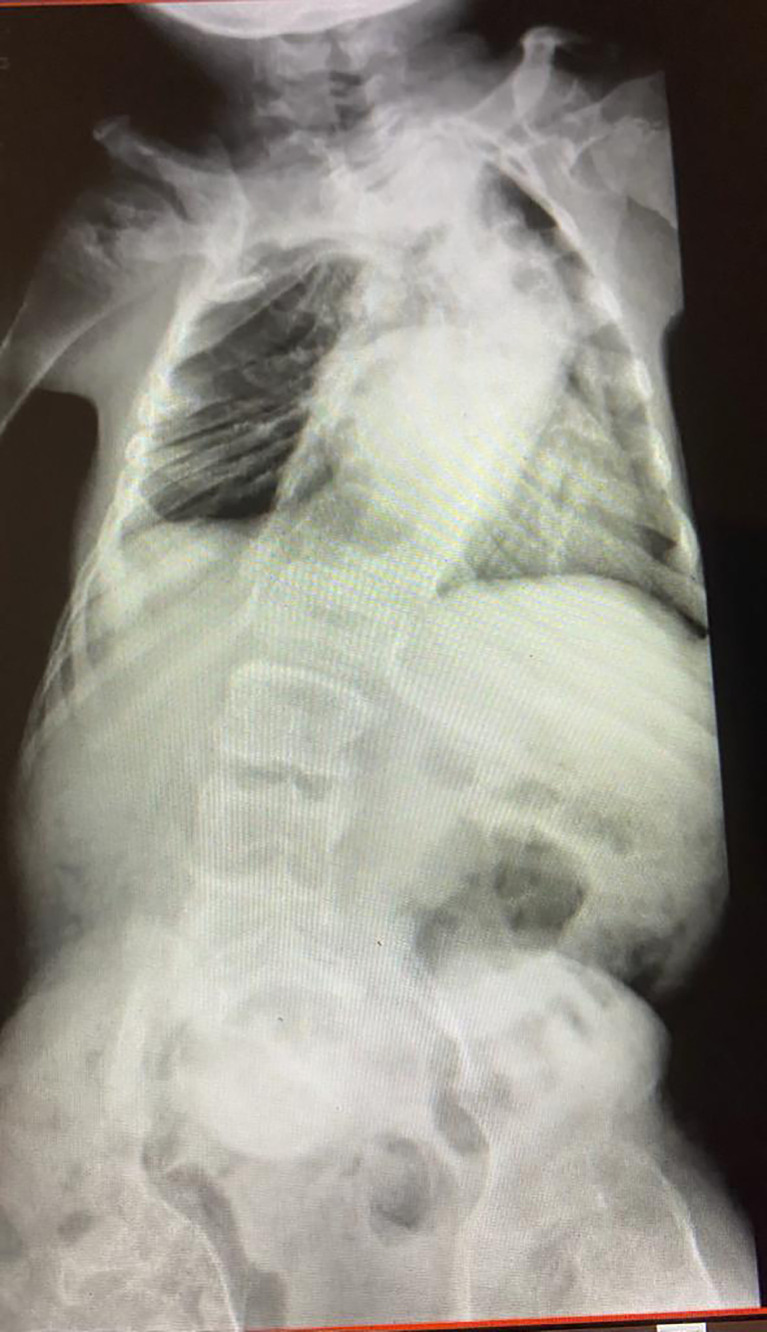
Whole spine X-ray of a patient with Osteogenesis Imperfecta and infected with COVID-19 showed severe scoliosis and Cobb’s angle of 70 in the thoracic curve.

9 (75%) of patients were less than 18 years, and 6 (50%) were males. 5 (41%) had kyphoscoliosis, 5 (41%) were wheelchair-bound. Pulmonary function tests were performed in only one patient. 6 (50%) were receiving bisphosphonate, 10 (83%) were receiving vitamin D, 7(58%) were receiving calcium, and 7(58%) had normal BMD. All patients did not require hospitalization and recovered with no complications. 11 (91%) patients had mild disease (fever, headache, myalgia, arthralgia, and cough). One patient was asymptomatic. 9 (75%) were eligible for vaccination. 5 (41%) were fully vaccinated. All patients had COVID-19 before or after partial vaccination (**Table 2**). **Figure 1** shows the spine X-ray of case 6, which had severe restrictive lung disease.

**Table 2:** Characteristics of patients with Osteogenesis Imperfecta infected with COVID-19 (n = 12).

Case	Age gender	Kyphoscoliosis	Use of pamidronate zoledronate	Use of Calcium	Use of Vitamin D	Bone mineral density	Functional status	Vaccination status	COVID19severity	Timing of COVID19	Outcome
	
Case1	10 years	NO	NO	NO	YES	Abnormal (Z -7.4)	Walking with assistance	Not vaccinated	Mild disease	Not applicable	Recovered
	male							Not eligible	Home isolated		
Case 2	12 years	YES	NO	YES	YES	Normal (Z 2)	Wheelchair bound	Not vaccinated	Mild disease	Not applicable	Recovered
	male							eligible	Home isolated		
Case 3	20 years	YES	NO	YES	YES	Abnormal (Z -2.7)	Wheelchair bound	Fully Vaccinated	Mild disease	6 days after 2^nd^ vaccine	Recovered
	male							(Astrazeneca)	Home isolated		
Case 4	19 years	NO	NO	YES	YES	Normal (Z -1.9)	Wheelchair bound	Partially Vaccinated	Mild disease	54 days after 1^st^ vaccine	Recovered
	female							(Pfizer)	Home isolated		
Case 5	5 years	NO	NO	NO	NO	Normal (Z 0.8)	Independent in walking and daily activities	Not vaccinated	Asymptomatic	Not applicable	Recovered
	male							Not eligible	Home isolated		
Case 6	16 years	YES	zoledronate	YES	YES	Abnormal (Z -2)	Wheelchair bound	Partially Vaccinated	Mild disease	COVID19	Recovered
	male	FVC 0.35L						(Pfizer)	Home isolated	Before vaccine	
Case 7	10 years	NO	zoledronate	NO	NO	Abnormal (Z -2.2)	Independent in walking and daily activities	Not vaccinated	Mild disease	Not applicable	Recovered
	male							Not eligible	Home isolated		
Case 8	16 years	YES	zoledronate	YES	YES	Abnormal (Z -2.5)	Walking with assistance	Fully Vaccinated	Mild disease	14 days after 1^st^ vaccine	Recovered
	female							(Pfizer)	Home isolated		
Case 9	16 years	NO	zoledronate	YES	YES	Normal (Z 0.6)	Independent in walking and daily activities	Fully Vaccinated	Mild disease	COVID19	Recovered
	female							(Pfizer)	Home isolated	Before vaccine	
Case 10	13 years	NO	Zoledronate	NO	YES	Normal (Z -0.7)	Wheelchair bound	Partially Vaccinated	Mild disease	COVID19	Recovered
	female							(Pfizer)	Home isolated	Before vaccine	
Case 11	18 years	NO	Pamidronate	YES	YES	Normal (Z -1.9)	Walking with assistance	Fully Vaccinated	Mild disease	COVID19	Recovered
	female							(Pfizer)	Home isolated	Before vaccine	
Case 12	15 Years	YES	NO	NO	YES	Normal (Z -0.2)	Independent in walking and daily activities	Fully Vaccinated	Mild disease	COVID19	Recovered
	female							(Pfizer)	Home isolated	Before vaccine	

## Discussion

In our cohort, all patients with OI had mild COVID-19, did not require hospitalization, and recovered without complications. Almost half of the patients develop COVID-19 before vaccination or after partial vaccination during the period of circulating SARS-CoV-2 variants of concern, predominately SARS-CoV-2 Delta virus (14). Nine out of 12 patients were less than 18 years old, 41% were chair bound, and 41% had kyphoscoliosis. 50% of patients received bisphosphonate, and 57% had normal BMN.

A multi-center study evaluated the extent of pulmonary dysfunction among patients with OI and found that patients with severe OI had significant restrictive lung disease even in the absence of kyphoscoliosis. The mean age of the study population was older than our study (15). Unfortunately, the extent of restrictive lung disease among patients with OI could not be evaluated in our research as pulmonary function tests were not performed except in one patient who had severe restrictive lung disease. At the same time, most of them were children and were not cooperative. In addition, patients with OI were not referred to the pulmonary service without respiratory complications. Chronic lung diseases are significant predictors of COVID-19 disease severity and mortality. Most studies evaluated patients with obstructive pulmonary diseases, interstitial lung diseases, and pulmonary vascular diseases with limited data on kyphoscoliosis and restrictive lung diseases (16). Only one case report described the fatal outcome of COVID-19 in a 57 years old male patient with severe restrictive lung disease secondary to advanced idiopathic kyphoscoliosis (17).

The use of bisphosphonate in patients with OI increased bone mineral density, reduced fractures, and improved functional activity (18–20). Recently, several studies described that fractures in adults infected with COVID-19 were associated with a worse outcome compared to non-COVID-19 patients. A meta-analysis showed that COVID-positive patients with hip fractures had significantly higher mortality than COVID-19 negative patients (21). Di Filippo compared the outcome of COVID-19 patients among patients with and without thoracic vertebral fractures retrospectively. COVID-19 patients with vertebral fractures were older, had co-morbidities, required hospitalization, and non-invasive ventilation compared to those without fractures. Although there was no significant mortality difference between COVID-19 patients with and without vertebral fractures, patients with severe fractures had significantly higher mortality than those with mild and moderate fractures (22). Di Filippo defined the osteo-metabolic phenotype of COVID-19 as hypocalcemia, hypovitaminosis D, and vertebral fractures described its poor outcome, and suggested therapeutic and preventive measures such as calcium, vitamin D, and anti-osteoporotic therapy (23). In our cohort of OI, more than two-thirds of patients were started on bisphosphonate since the time of their diagnosis to reduce their fracture risks. Based on animal studies, bisphosphonate was found to have anti-inflammatory and immune-modulating effects, which may improve the outcome of SARS-CoV-2 pneumonia (24). Several observational retrospective case-controlled studies showed conflicting results regarding the impact of bisphosphonate on the development of adult patients hospitalized with COVID-19 pneumonia (25–27). One retrospective study showed that patients on parenteral zoledronate had a low incidence of COVID-19 after adjustment for different confounding factors such as age and comorbidities (25). While another two retrospective studies did not show that prior use of bisphosphonate did improve the outcome and progression to severe disease in COVID-19 adult patients (26, 27). It is worth emphasizing that these studies evaluated the prior use of bisphosphonate and its effect on COVID-19 disease severity without describing the degree of BMD and the presence of fractures.

Patients with rare genetic diseases were adversely affected during the COVID-19 pandemic, with limited access to medical care (28). Brizola E et described their experience in Italy in providing the Phone calls Helpline initiative to follow patients with rare bone diseases remotely during the COVID-19 pandemic. It was successful in answering their concerns and providing them with valuable educational information (29). In our study, among patients with OI eligible for vaccination, 66% of them received two doses of COVID-19 vaccination, though the vaccination of children in Saudi Arabia was only approved on 27th June 2021 (30). In Saudi Arabia, patients with OI were closely followed by the endocrinology service in tertiary care centers. In addition, there was no delay in administering parenteral zoledronate to these patients in the medical day units despite significant hospital limitations benefits during the COVID-19 pandemic. Adult patients with OI that do not require bisphosphonate and orthopedic procedures are transferred to primary care hospitals for future follow-up in different towns. It was challenging to reach their primary physicians to evaluate their status during the COVID-19 pandemic.

Our study was mainly descriptive for the outcome of COVID-10 patients with OI, predominantly children with mild disease. It was limited by the retrospective design and small sample, which did not evaluate predictors for severe COVID-19 illness in patients with OI. While all patients had results of BMN, biochemical parameters including serum calcium and vitamin D levels were not available for all patients. The lack of pulmonary function tests in most patients precludes the objective assessment of restrictive lung disease and its severity, being a significant risk factor for severe COVID-19. Based on our study design, we could not demonstrate whether the use of bisphosphonate could improve the outcome of COVID-19 patients with OI by reducing the rate of fractures and improving BMN.

## Conclusions

This is the largest cohort of COVID-19 patients with OI described in the literature. Unlike other patients with genetic diseases, patients with OI in our study recovered from COVID-19 without complications. Young age and mild disease most likely contributed to the successful outcome. Although half of the patients with OI received bisphosphonate and had normal BMD, our study was not designed to evaluate the impact of bisphosphonate use on COVID-19 severity. While the prior use of bisphosphonate did not improve the outcome of COVID-19 adult patients, age and comorbidities were the most critical risk factors for severe COVID-19 pneumonia. Future studies are needed to evaluate the effect of bisphosphonate use and the degree of BMD on the prognosis of hospitalized COVID-19 adult patients. In addition, the outcome of COVID-19 in adult patients requires further investigation.

## Data Availability Statement

The raw data supporting the conclusions of this article will be made available by the authors, without undue reservation.

## Ethics Statement

The studies involving human participants were reviewed and approved by Institutional Research Board, King Faisal Specialist Hospital And Research Center, Jeddah, Saudi Arabia. Written informed consent from the participants’ legal guardian/next of kin was not required to participate in this study in accordance with the national legislation and the institutional requirements.

## Author Contributions

All authors contributed to the study concept, study design, literature review, data collection, data analysis, reviewing, editing, and writing the manuscript. All authors contributed to the article and approved the submitted version.

## Funding

This work was funded by King Faisal Specialist Hospital and Research Center, Jeddah, Kingdom of Saudi Arabia.

## Conflict of Interest

The authors declare that the research was conducted in the absence of any commercial or financial relationships that could be construed as a potential conflict of interest.

## Publisher’s Note

All claims expressed in this article are solely those of the authors and do not necessarily represent those of their affiliated organizations, or those of the publisher, the editors and the reviewers. Any product that may be evaluated in this article, or claim that may be made by its manufacturer, is not guaranteed or endorsed by the publisher.
